# Promyelocytic Leukemia (PML) gene regulation: implication towards curbing oncogenesis

**DOI:** 10.1038/s41419-019-1889-2

**Published:** 2019-09-10

**Authors:** Neerajana Datta, Saimul Islam, Uttara Chatterjee, Sandip Chatterjee, Chinmay K. Panda, Mrinal K. Ghosh

**Affiliations:** 1 Cancer Biology and Inflammatory Disorder Division, Council of Scientific and Industrial Research-Indian Institute of Chemical Biology (CSIR-IICB), TRUE Campus, CN-6, Sector–V, Salt Lake, Kolkata-700091 & 4, Raja S.C. Mullick Road, Jadavpur, Kolkata, 700032 India; 2 Department of Oncogene Regulation, Chittaranjan National Cancer Institute, 37, S.P. Mukherjee Road, Kolkata, 700026 India; 3 Division of Neurosurgery, Division of Pathology, Park Clinic, 4, Gorky Terrace, Kolkata, 700017 India

**Keywords:** Transcription, Oncogenesis

## Abstract

Dysregulation of PML, a significant tumor suppressor is linked with cancers of different histological origins, with a decreased expression observed with a higher tumor grade. This necessitates studying the mechanisms to maintain a stable expression of PML. However much less is known about the transcriptional regulation of PML, more so in the context of breast carcinoma. ERβ has emerged as a critical factor in understanding breast cancer, especially since a huge proportion of breast cancers are ERα^−^ and thus insensitive to tamoxifen therapy. This study aims to uncover an unidentified mechanism of PML gene regulation and its stabilization in breast cancer *via* ERβ signalling and the impact on cellular apoptosis. We found that clinical expression of PML positively correlates with that of ERβ both in normal and breast carcinoma samples and inversely correlates with markers of cellular proliferation, hinting towards a possible mechanistic interdependence. Both mRNA and protein expression of PML were increased in response to ERβ overexpression on multiple human breast cancer cell lines. Mechanistically, luciferase reporter assays and chromatin-immunoprecipitation assays demonstrated that ERβ can interact with the PML promoter *via* ERE and AP1 sites to enhance its transcription. ERβ induced stable PML expression causes a decline of its target protein Survivin and simultaneously provides a stable docking platform leading to stabilisation of its target Foxo3a, further causing transcriptional upregulation of pro-apoptotic factors p21 and p27. Immunohistochemical analyses of cancer and normal breast tissues and functional assays conducted corroborated the findings. Collectively, our study identifies ERβ signalling as a novel mechanism for PML gene regulation in ERα^−^ breast cancer. It also reveals bi-directional downstream effect in which ‘ERβ-PML-(Foxo3a/Survivin)’ network acts as a therapeutic axis by suppressing cellular survival and promoting cellular apoptosis in breast carcinoma.

## Introduction

Promyelocytic Leukemia Protein (PML) is an essential component of PML Nuclear bodies (PML-NBs) where it plays a vital role in their formation and stability. PML-NBs act as cellular organizing centers for the coordinated regulation of various processes such as transcriptional regulation, post-translational modifications, DNA replication, apoptosis, senescence, cell cycle regulation and DNA damage repairs^1^. Hence, since its discovery PML is implicated in playing a role in carcinogenesis and more often than not vouched as a tumor suppressor. Histochemical analyses of clinical samples have shown PML to be downregulated in many cancer types such as that of breast, CNS, colon, prostate and Non-Hodgkin’s Lymphoma^2^. These studies highlight an important role for PML in tumor suppression; however, the mechanisms underlying the loss of PML are largely unknown.

The largest part of studies on PML regulation is conducted at the post-translation level, SUMOylation, phosphorylation and ubiquitination being the chief contributors^3^. On the contrary, much less is known about the transcriptional regulation of PML. The noted one is the interferon and TNFα induced transcriptional upregulation of PML mediated by STATs, which occupy the ISRE (induced by IFNα/β) and GAS (IFNγ activated) sites on PML promoter^4–7^. IL-6 also enhances PML transcription *via* NF-кβ and JAK/STAT pathway^8^. Activated Ras mediated transformation of MEFs also induces PML in a p53-dependent manner^9,10^.

Breast cancer (BCa) is the second most common cancer and the most common cancer among women in the world^11^. Though mostly diagnosed based upon the presence or absence of three receptors: ERα, PR and HER-2, ~15–20% of all types of BCa in women do not express these receptors and, are, thus, defined as triple-negative BCa (TNBC) and are hence insensitive to hormone responsive treatments and frequently undergo local or systemic relapse^12^. Additionally, 30–40% of ERα^+^ patients receiving adjuvant tamoxifen therapy also eventually relapse^13,14^. These indicate the need to identify new molecular signatures along with their prospective validation to derive novel therapeutic strategies.

One such factor could be ERβ. Although present in smaller quantity as compared to ERα, ERβ is found in about 70% of BCa^15,16^. As opposed to its alpha isoform, ERβ is known for its anti-proliferative actions^17,18^. ERβ1 positivity was associated with significantly better survival in patients with double-negative (ER^−^/PR^−^) or triple-negative tumors. Furthermore, ERβ expression in ERα^+^/ERβ^+^ breast tumors is associated with a favorable response to adjuvant tamoxifen therapy^19^. The ratio between ERβ and ERα is high in normal glands, and decreases significantly in proliferative lesions^20^. This collected information directs to the possible role of ERβ in restoring the tumor suppression mechanism in BCa. In this study, we dissect the mechanisms underlying transcriptional regulation of PML in response to ERβ. Upregulation of PML leads to decrease in downstream target Survivin along with increased stability of tumor suppressor Foxo3a, which in turn leads to upregulation of its transcriptional targets p21, and p27, thus leading to apoptosis and curbing oncogenesis.

## Results

### Concomitant loss of ERβ and PML in human breast cancer samples

**Fig. 1:**
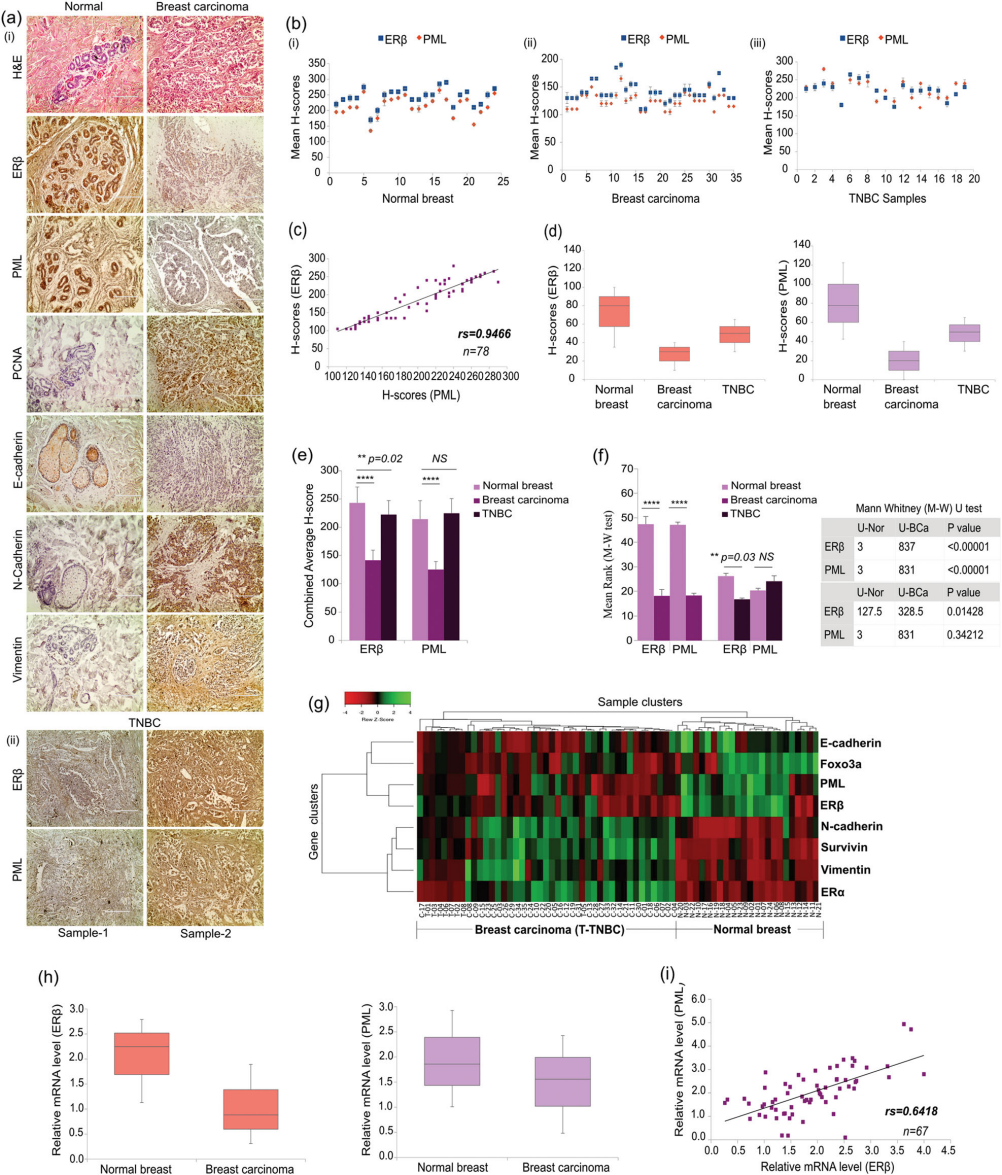
Concomitant loss of ERβ and PML gene expression and both share a strong positive correlation in human breast tissue samples. **a (i)** Representative IHC images of the candidate proteins along with respective H&E staining in human BCa and adjacent normal breast tissue samples and **(ii)** IHC images of ERβ and PML in two representative TNBC samples (Samples 1 and 2). All images are taken at ×200 magnification. **b** Scatter-plot representation of the mean H-scores of ERβ and PML in (i) adjacent normal breast (*n* = 24), (ii) breast carcinoma (ER^+^) (*n* = 35) and (iii) TNBC (*n* = 19) tissues. **c** Depiction of correlation coefficient (*r*_*s*_) between mean H-scores of ERβ and PML estimated from IHC images of both normal breast and BCa tissues combined. **d** Box-plots depicting distribution of H-scores of ERβ and PML, in normal breast, BCa and TNBC samples. **e** Comparison of combined average H-scores of ERβ and PML. **f** Graphical representation of mean ranks of the observed individual H-scores of ERβ and PML as obtained through calculations from M–W *U*-test (left). Table displaying the calculated Mann–Whitney *U*-test values for the H-scores of each observed protein is on the right. **g** Heat map of expression patterns of 8 genes from 24 normal (N) and 43 BCa (C) samples that includes 8 TNBC (T) samples, as obtained from qRT-PCR data (w.r.t GAPDH), displayed on a scale from green (low) to red (high). **h** Box-plots depicting relative gene expression of ERβ and PML (w.r.t GAPDH) in normal breast and BCa samples. **i** Depiction of correlation coefficient (*r*_*s*_) between mean relative expressions of ERβ and PML genes as quantified from qRT-PCR data of both normal breast and BCa tissues combined. The error bars represent the mean (±) s.d. of independent two-tailed Student’s t-tests, where *P* < 0.0001 is represented as **** for highly significant

To explore any possible connection between ERβ and PML in BCa we conducted immunohistochemical analyses in BCa (*n* = 53) [including TNBC (*n* = 19)] and adjacent normal tissue samples (*n* = 24). ERβ and PML show elevated expressions in normal tissues and a sharp decline in BCa samples. As expected, N-cadherin, Vimentin and PCNA show enhanced expressions in carcinoma samples as opposite to E-cadherin which demonstrates a weakened expression (Fig. 1a (i), Fig. S1a). Interestingly, a subset of TNBC samples shows a heightened expression of ERβ along with PML, as opposed to the bulk of ERα^+^ BCa samples. However, the TNBC samples maintain an overall positive correlation between ERβ and PML (Fig. 1a (ii), Fig. S1b). Upon quantification by H-scoring, both ERβ and PML depict significant differences in staining intensities between BCa and normal samples (Fig. 1b) and Spearman’s rank correlation confirms that H-scores of ERβ and PML bear a strong positive correlation (*rs* = 0.946) (Fig. 1c). Furthermore, the distribution and the difference between mean H-scores of these proteins are statistically significant in BCa samples as compared to the normals, as also predicted by Mann–Whitney U-test (Fig. 1d, e, f).

qRT-PCR analyses conducted in the RNA isolated from the same set of tissue samples showed a unique grouping of ERβ and PML that shared the strongest correlation. Sample analyses too demonstrated a distinct clustering, with the normal and BCa samples on two ends of the spectrum (Fig. 1g). The distribution of ERβ and PML gene expression as calculated from normalized Ct values show a distinct difference between the BCa and normal tissue samples (Fig. 1h) and Spearman’s rank analysis put a moderately strong positive correlation (*rs* = 0.642) between the two (Fig. 1i). Altogether, results indicate that ERβ and PML show a concomitant loss while maintaining positive correlation in human breast tissue samples.

### ERβ regulates the expression level of PML protein in breast cancer cells

**Fig. 2:**
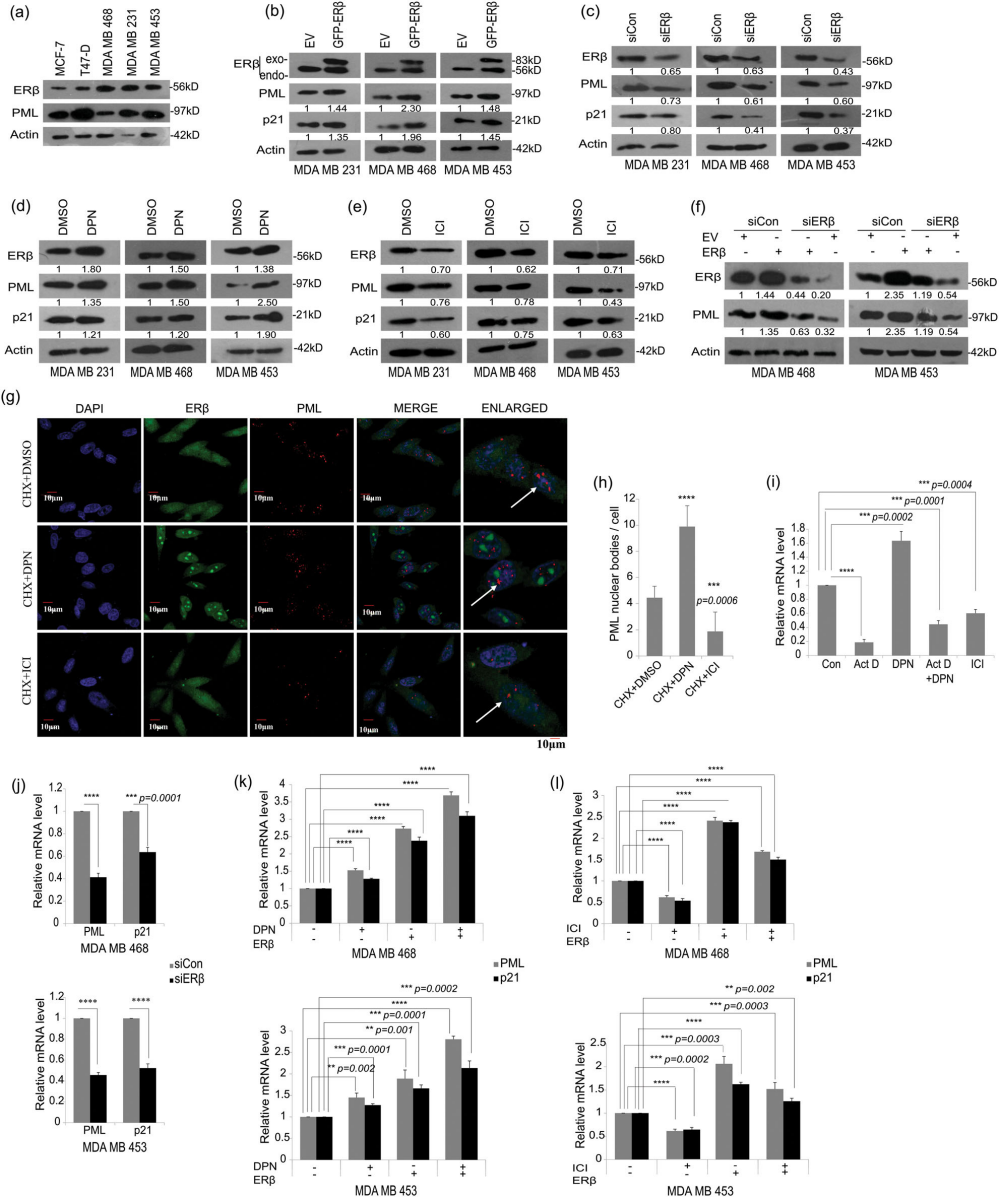
ERβ positively regulates PML expression in breast cancer (BCa). **a** Immunoblot shows differential expression of ERβ and PML in multiple BCa cell lines. Three TNBC cell lines as depicted in the subsequent figures were used in this study. **b** Immunoblot analysis of cells transfected with either GFP-ERβ or the empty vector and treated with DPN or DMSO and harvested at a total of 48 hr post transfection. Densitometric analyses relative to loading control Actin are mentioned below the blots. p21 acts as positive control for ERβ overexpression. **c** Immunoblot analysis of cells transfected with either scrambled siRNA or siRNA against ERβ and harvested 48 hr post transfection. Immunoblot analysis of cells treated with **d** DPN (10 nM) or **e** ICI (1 µM) or DMSO 24 hr post treatment. **f** Immunoblot analysis of cells transfected with either WT-ERβ or the empty vector, treated with DPN/DMSO and further transfected with either scrambled siRNA or siRNA against ERβ. **g** Expression of ERβ and PML in MDA MB 468 cells treated with either DPN or ICI or DMSO control in combination with CHX, detected by immuno-cytochemistry. All images were taken at ×120 maginification. Scale bars, 10 μm. All experiments were performed in triplicates, *n* = 3. **h** Quantification of average number of PML nuclear bodies per MDA MB 468 cell treated with DPN or ICI in combination with CHX. PML-NBs were counted out of multiple randomly chosen fields from three biologically repeated experiments. **i** Relative mRNA expression (w.r.t 18 s rRNA) of PML assessed in MDA MB 468 cells treated with DPN or ICI, with actinomycin D (ActD) for 4 hr either alone or prior to DPN treatment. **j** qRT-PCR analysis of cells transfected with either scrambled siRNA (siCon) or siRNA against ERβ. **k** qRT-PCR analysis of cells transfected with either GFP-ERβ and/or treated with DPN. **l** qRT-PCR analysis of cells transfected with either empty vector or GFP-ERβ along with or without ICI. The error bars represent the mean (±) s.d. of independent two-tailed Student’s *t*-tests, where *P* < 0.0001 is represented as **** for highly significant

To investigate the possible mechanistic interplay between PML and ERβ in BCa, we checked the endogenous expression of ERβ in multiple BCa cell lines. A prominent expression was seen in the three TNBC cell lines, which were hence used in further studies (Fig. 2a). To study the correlation *in vitro*, we overexpressed ERβ and activated it with ERβ specific ligand DPN^21,22^. We observed an enhanced expression of PML upon ERβ activation in HEK293 as well as in multiple TNBC cell lines. p21 is a reported target gene of ERβ action^23^ (Fig. 2b, Fig. S2a). On depleting endogenous ERβ we observed a sharp decline in PML expression (Fig. 2c). PML showed a sharp increase on treating the cells with DPN and plummeted on treatment with anti-estrogen ICI^24–26^ (Fig. 2d, e, Fig. S2b). In a combinatorial approach experiment, ERβ knockdown caused a decrease in PML expression, abolishing the effect of ERβ overexpression and its ligand dependent activation in TNBC cell lines (Fig. 2f). As observed from immuno-cytochemical studies, ERβ has a diffused presence all over the nucleus and cytoplasm, while interestingly, in the presence of agonist, it significantly accumulates in numerous nuclear foci. Simultaneously, DPN caused a visible increase in the number of PML-NBs that remain distributed throughout the nucleus whereas treatment with ICI shows a decline in their number (Fig. 2g, h). Taken together, we observe that ligand activated and/or overexpressed ERβ enhances PML expression.

### ERβ transcriptionally regulates PML gene expression in breast cancer cells

For our mRNA studies we designed two qRT-PCR primer sets for PML spanning two different exon–exon junctions (Fig. S3a). Overexpression of ERβ led to significant upregulation of PML, as validated by both the primer sets (Fig. S3b). Set 1 is used for further studies as it would cover transcripts of each of the PML isoforms I–VI while Set 2 is specific only to PML-I mRNA. Actinomycin D caused a sharp fall in PML mRNA levels, similar to treatment with ICI and nullifies the effect of DPN (Fig. 2i). Since a combination of two siERβ oligonucleotides led to a better depletion of endogenous ERβ level in comparison to single siRNA’s (Fig. S3c), this strategy is used for ERβ knockdown. ERβ depletion led to significant reduction in PML mRNA level (Fig. 2j). An increase in PML mRNA expression was observed on overexpressing ERβ alone or on treatment with DPN that augments further when ERβ overexpression is coupled with DPN treatment (Fig. 2k). Overexpression and activation of ERβ also leads to an increase in other pro-apoptotic factors and known targets of ERβ action, *viz*, p21, p27, Bim and Foxo3a (Fig. S3d, e). Conversely, treatment with ICI led to almost two-fold decrease in PML mRNA expression (Fig. 2l). These results imply that ERβ regulates both mRNA expression and protein level of PML in multiple TNBC cell lines.

### ERβ regulates PML promoter activity

**Fig. 3:**
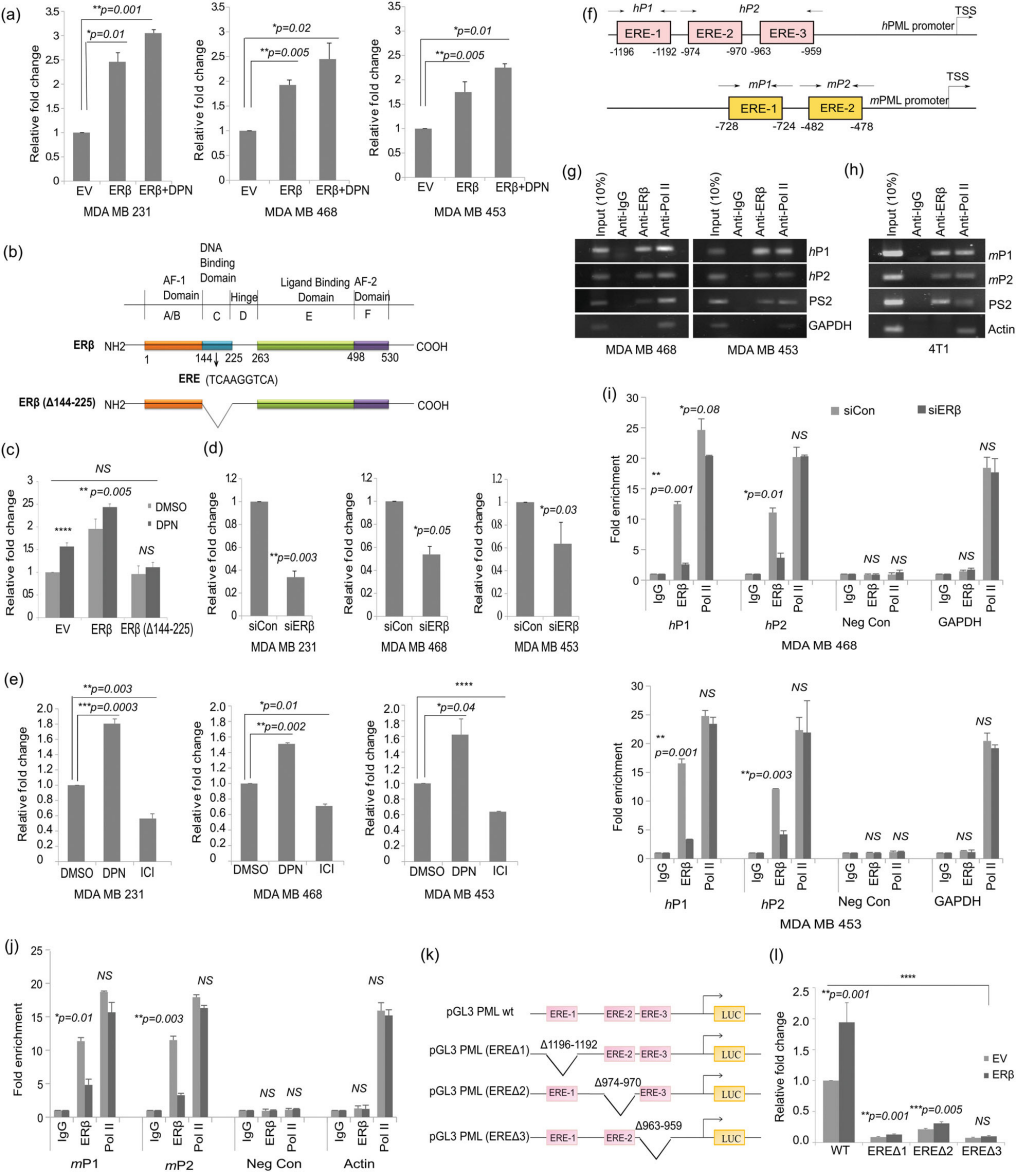
ERβ regulates PML promoter activity. **a** Luciferase activity of PML promoter in cell lines co-transfected with WT-ERβ, pGL3-PML-prom(human), and pRL-TK (Renilla luciferase construct), and treated with either DMSO or DPN. The figure represents relative fold change in luciferase readings, normalized against Renilla reporter activity. **b** Schematic representation of wild type ERβ gene mentioning all its domains and its mutant construct pcDNA ERβ (Δ144-225), with its DNA binding domain (DBD) deleted. ERβ DBD can occupy the ERE sites (consensus sequence TCAAGGTCA) on ERβ target promoters. **c** Luciferase activity of PML promoter in MDA MB 468 cells transfected with either WT-ERβ or the mutant construct pcDNA ERβ (Δ144-225), along with pGL3-WT-PML-prom(human) and pRL-TK. **d** Luciferase activity of PML promoter co-transfected with either scrambled siRNA or siRNA against ERβ, pGL3-PML-prom(human) and pRL-TK. **e** Luciferase activity of PML promoter co-transfected with pGL3-PML-prom(human) and pRL-TK and treated with DMSO, DPN or ICI. **f** Schematic representation of human (−1447 to +250) and mouse (−800 to TSS) PML promoters containing ERE sites. Chromatin immunoprecipitation (ChIP) assay performed using the indicated antibodies in **g** human MDA MB 468 and MDA MB 453 and **h** mouse 4T1 cells. RNA Polymerase II and IgG served as positive and negative controls respectively and PS2 promoter served as the control for ERβ binding. GAPDH and Actin promoters served as positive controls for Pol II in human and mouse ChIP assays respectively. ChIP assay performed on **i** MDA MB 468 and MDA MB 453 cells and **j** 4T1 cells transfected with either scrambled siRNA or with siRNA against ERβ. ‘Neg Con’ stands for non-genomic intragenic regions serving as negative control for the same. **k** Schematic representation of the human WT pGL3 PML promoter and the three ΔERE deletion constructs. **l** Luciferase activity measured in MDA MB 468 cells co-transfected with either WT ERβ along with pGL3-WT-PML-prom(human) or its deletion constructs and pRL-TK. Data are normalized to Renilla luciferase activity and represented as fold activity with respect to control cells. Error bars represent mean (±) s.d. calculated from three independent experiments. *P* < 0.0001 is represented as **** for highly significant and NS denotes ‘not significant’

We cloned human and mouse PML promoters into pGL3 vector. Ligand independent ERβ overexpression led to an increase in human PML luciferase activity that augmented when treated with DPN (Fig. 3a, Fig. S4a). DPN can also directly activate two ERβ reporters: 3 × -ERE-TATA-luc and p21-luc in TNBC cell lines as opposed to HEK293, but more interestingly, ERβ unaided by a ligand is often able to surpass the sole response of DPN (Fig. S4c, d). Not surprisingly, ERβ when aided by a ligand has the highest response on both the reporters. We further generated an ERβ mutant construct (Δ144-225) that lacks the ERβ DNA binding domain (DBD: amino acids 144–225) and introduced it or WT-ERβ in our overexpression system to study PML promoter activity (Fig. 3b). The luciferase activity of the mutant remained unchanged from its empty vector control, thus indicating that ERβ binding on PML promoter is direct (Fig. 3c). Conversely, depletion of endogenous ERβ led to a decline in PML luciferase activity (Fig. 3d, Fig. S4b). This effect was further validated when DPN led to an increase in PML promoter activity and ICI caused a decline (Fig. 3e). Here too we observed a heightened response of unliganded ERβ as compared to DPN treatment on PML promoter activity. We establish so far that ERβ increases PML expression by transcriptional activation of its promoter.

### ERβ associates with chromatin and regulates PML promoter

ERβ can transcriptionally regulate its target genes by either directly occupying Estrogen Response Element (ERE) sites on target promoters or indirectly by tethering to co-activators such as AP-1^27,28^. As analyzed from Alggen PROMO and Eukaryotic Promoter database three half ERE sites and three AP-1 sites were observed upstream of TSS in human PML promoter (−1447 to +250 bp). Two half ERE sites were observed in mouse PML promoter in a span of 800 bp upstream of TSS (Fig. 3f, Fig. S5a).

The chromatin immunoprecipitation (ChIP) data demonstrated that endogenous ERβ, without any agonist treatment, binds to PML promoter *in vivo* by tethering to both ERE and AP-1 sites (Fig. 3g, h, Fig. S5b). Under ERβ knockdown conditions, ERβ occupancy of endogenous PML promoter was sharply reduced (Fig. 3i, j). To decipher the strength of binding at each of the ERE sites, we generated three deletion mutants (Fig. 3k). Luciferase activities of mutant PML promoters were significantly reduced, the effect being most prominent in EREΔ1 and EREΔ3. Also, the increase in PML promoter activity due to ERβ overexpression was abrogated in the case of EREΔ3 (Fig. 3l). However a much less decrease in PML promoter activity was observed on deletion of AP-1 sites as compared to that of ERE sites, with maximum reduction seen in AP1Δ1 (60%) which is lesser than the minimum reduction observed in EREΔ2 (80%) (Fig. S5c). Furthermore there is a minimal but significant increase in PML promoter activity on exogenous ERβ overexpression in all the AP-1 deletion mutants, which is far less than that observed with WT-PML promoter. These findings allow us to remark that unliganded ERβ can directly interact with PML promoter, via its DBD, occupying the ERE and AP-1 sites. ERβ binding on ERE sites is more effective in transcriptional activation of the PML promoter as compared to the AP-1 sites.

### Foxo3a loss and Survivin gain in breast cancer samples is correlated to loss of ERβ and PML

**Fig. 4:**
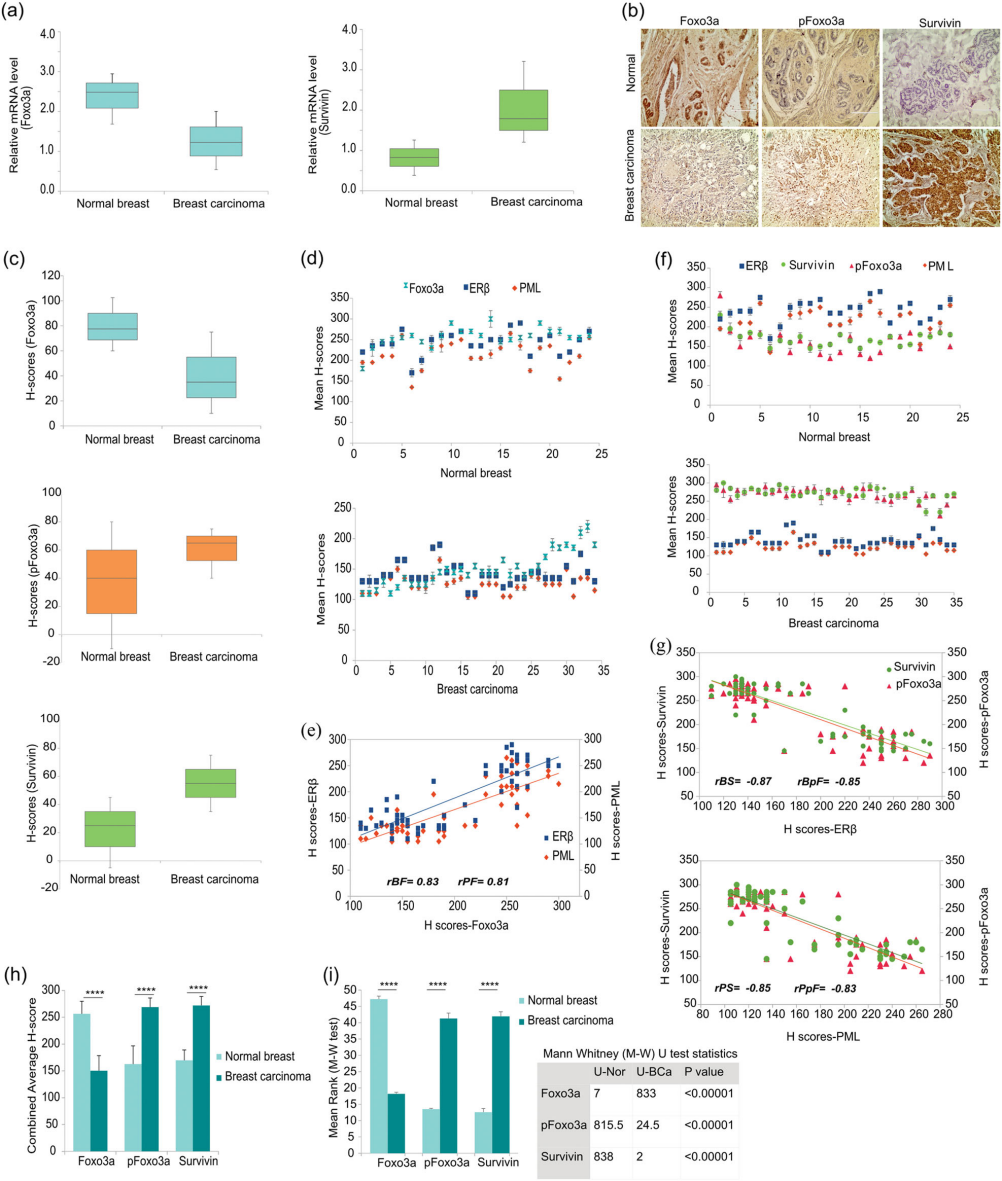
ERβ shares a positive correlation with Foxo3a and inversely correlates with Survivin in human breast cancer samples. **a** Box-plots depicting relative gene expression of Foxo3a and Survivin (w.r.t GAPDH) in the same set of normal breast and BCa samples, as used in Fig. 1. **b** Representative IHC images of the candidate proteins Foxo3a, pFoxo3a and Survivin of the human BCa and adjacent normal breast tissues (the same samples used in Fig. 1a). All images are taken at ×200 magnification. **c** Box-plots depicting distribution of H-scores of Foxo3a, pFoxo3a and Survivin, in normal breast (*n* = 24) and BCa (*n* = 35) samples. Scatter-plot representation of the mean H-scores of **d** ERβ, PML and Foxo3a and **f** ERβ, PML, pFoxo3a and Survivin in adjacent normal breast and BCa tissues. Depiction of correlation coefficient (*rs*) between mean H-scores of **e** ERβ-Foxo3a-PML **(g)** Survivin-ERβ-pFoxo3a (top) and Survivin-PML-pFoxo3a (bottom) estimated from IHC images of both normal breast and BCa tissues combined. **h** Comparison of combined average H-scores of Foxo3a, pFoxo3a and Survivin. **i** Graphical representation of mean ranks of the observed individual H-scores of Foxo3a, pFoxo3a and Survivin as obtained through calculations from M–W U-test (left). Table displaying the calculated Mann–Whitney U-test values for the H-scores of each observed protein is on the right. The error bars represent the mean (±) s.d. of independent two-tailed Student’s *t*-tests. *P* < 0.0001 is represented as **** for highly significant

A previous work from our group had elucidated how stabilized PML acts as a scaffolding platform for activated Akt (pAKT) and its phosphatase (PHLPP2) inside the nucleus leading to de-phosphorylation and proteasomal degradation of Akt and hence stabilization of Foxo3a, in prostate cancer^29^. On the other hand PML is also known to induce apoptosis by down-regulation of Survivin, where it is seen that PML represses Survivin protein and mRNA expression in MEFs^30^. We speculated if these effects hold true in BCa. Gene expression analyses conducted on the same set of normal and BCa samples as used in Fig. 1, showed a higher expression of Survivin in BCa samples as opposed to that of Foxo3a which is abundantly overexpressed in normal breast samples (Fig. 4a). IHC analyses of BCa patient samples displayed a very low expression of Foxo3a, whereas pFoxo3a^S253^ (activated Akt phosphorylates Foxo3a at Ser-253 leading to its inactivation and degradation) showed distinct elevated staining. The reverse was observed in the normals. Survivin displayed marked elevation in BCa samples as compared to the normals (Fig. 4b, Fig. S1c). The box-plot distribution (Fig. 4c) and H-score analysis (Fig. 4d) proved that ERβ, PML and Foxo3a share a similar trend in both BCa, as well as in normal samples, whereas ERβ and PML share an opposing trend with pFoxo3a^S253^ and Survivin (Fig. 4f). Spearman’s Correlation drawn on H-scores, showed that ERβ and PML share a strong positive correlation with Foxo3a (*r*_*s*_ *=* 0.83 and 0.81, respectively) (Fig. 4e). A strong negative correlation exists between ERβ-Survivin (*r*_*s*_ = −0.87), ERβ-pFoxo3a^S253^ (*r*_*s*_ *=* −0.85), PML-Survivin (*r*_*s*_ *=* −0.85) and PML-pFoxo3a^S253^ (*r*_*s*_ *=* −0.83) (Fig. 4g). The combined average H-scores (Fig. 4h) and the difference in the H-scores of Foxo3a, pFoxo3a^S253^ and Survivin between normal and the BCa samples was also statistically significant as analyzed by Mann–Whitney U-test (Fig. 4i). The expression of “ERβ-PML-(Foxo3a/Survivin)” signaling pathway components has been summarized in Tables 1 and 2. From these results we deduced that ERβ-PML signalling might give a stronghold on the expression of Foxo3a and Survivin in BCa.

**Table 1:** Correlation coefficients between immuno-histochemistry scores of the ERβ-PML-(Foxo3a/Survivin) signaling pathway components in normal human breast and breast cancer tissues

			PML			Foxo3a			pFoxo3a			Survivin		
			T	N	BCa	T	N	BCa	T	N	BCa	T	N	BCa
ERβ	T	*r*_*s*_	0.980			0.839			−0.853			0.874		
		*P*	0.029			0.002			****			****		
	N	*r*_*s*_		0.948			0.787			−0.546			−0.537	
		*P*		0.002			0.001			****			****	
	BCa	*r*_*s*_			0.844			0.884			−0.697			−0.722
		*P*			****			0.031			****			****
PML	T	*r*_*s*_				0.815			−0.835			−0.856		
		*P*				0.002			0.015			0.004		
	N	*r*_*s*_					0.744			−0.720			−0.709	
		*P*					0.035			0.039			0.022	
	BCa	*r*_*s*_						0.414			0.320			0.860
		*P*						0.132			0.210			0.001
Foxo3a	T	*r*_*s*_							−0.903			−0.938		
		*P*							0.003			****		
	N	*r*_*s*_								−0.486			−0.676	
		*P*								****			****	
	BCa	*r*_*s*_									−0.600			−0.613
		*P*									****			****

T-Total samples, N-Normal human breast samples (*n* = 24), BCa-Human breast cancer samples (*n* = 35). *r*_*s*_- Pearson’s Correlation Coefficient, *P*- Student’s *t*-Test, significant at *P* < 0.05

**Table 2:** Summary of the corresponding immune-histochemistry expression of ERβ-PML-(Foxo3a/Survivin) signaling pathway components in human normal breast (*n* = 24) and breast cancer tissues (*n* = 35)

				Normal	Foxo3a-Low	Foxo3a-High	Total	Normal	Foxo3a-Low	Foxo3a-High	Total
				**PML-Low**	0	0	0	**ERβ-Low**	0	0	0
				**PML-High**	1	23	24	**ERβ-High**	0	24	24
				**Total**	1	23	24	**Total**	0	24	24
**Normal**	**PML-Low**	**PML-High**	**Total**								
**ERβ-Low**	0	0	0	**Breast Cancer**	**Foxo3a-Low**	**Foxo3a-High**	**Total**	**Breast Cancer**	**Foxo3a-Low**	**Foxo3a-High**	**Total**
**ERβ-High**	1	23	24	**PML-Low**	21	1	27	**ERβ-Low**	16	11	27
**Total**	1	23	24	**PML-High**	13	0	8	**ERβ-High**	6	2	8
				**Total**	34	1	35	**Total**	22	13	35
**Breast Cancer**	**PML-Low**	**PML-High**	**Total**	**Normal**	**Survivin-Low**	**Survivin-High**	**Total**	**Normal**	**Survivin-Low**	**Survivin-High**	**Total**
**ERβ-Low**	27	0	27	**PML-Low**	1	4	5	**ERβ-Low**	0	0	0
**ERβ-High**	7	1	8	**PML-High**	0	19	19	**ERβ-High**	5	19	24
**Total**	34	1	35	**Total**	1	23	24	**Total**	5	19	24
				**Breast Cancer**	**Survivin-Low**	**Survivin-High**	**Total**	**Breast Cancer**	**Survivin-Low**	**Survivin-High**	**Total**
				**PML-Low**	0	0	27	**ERβ-Low**	0	27	27
				**PML-High**	34	1	8	**ERβ-High**	0	8	8
				**Total**	34	1	35	**Total**	0	35	35

IHC expression in the range 0–150 is considered as Low and that between 151 and 300 is considered as High

### Downregulation of Foxo3a and upregulation of Survivin is the result of ERβ-PML signaling

**Fig. 5:**
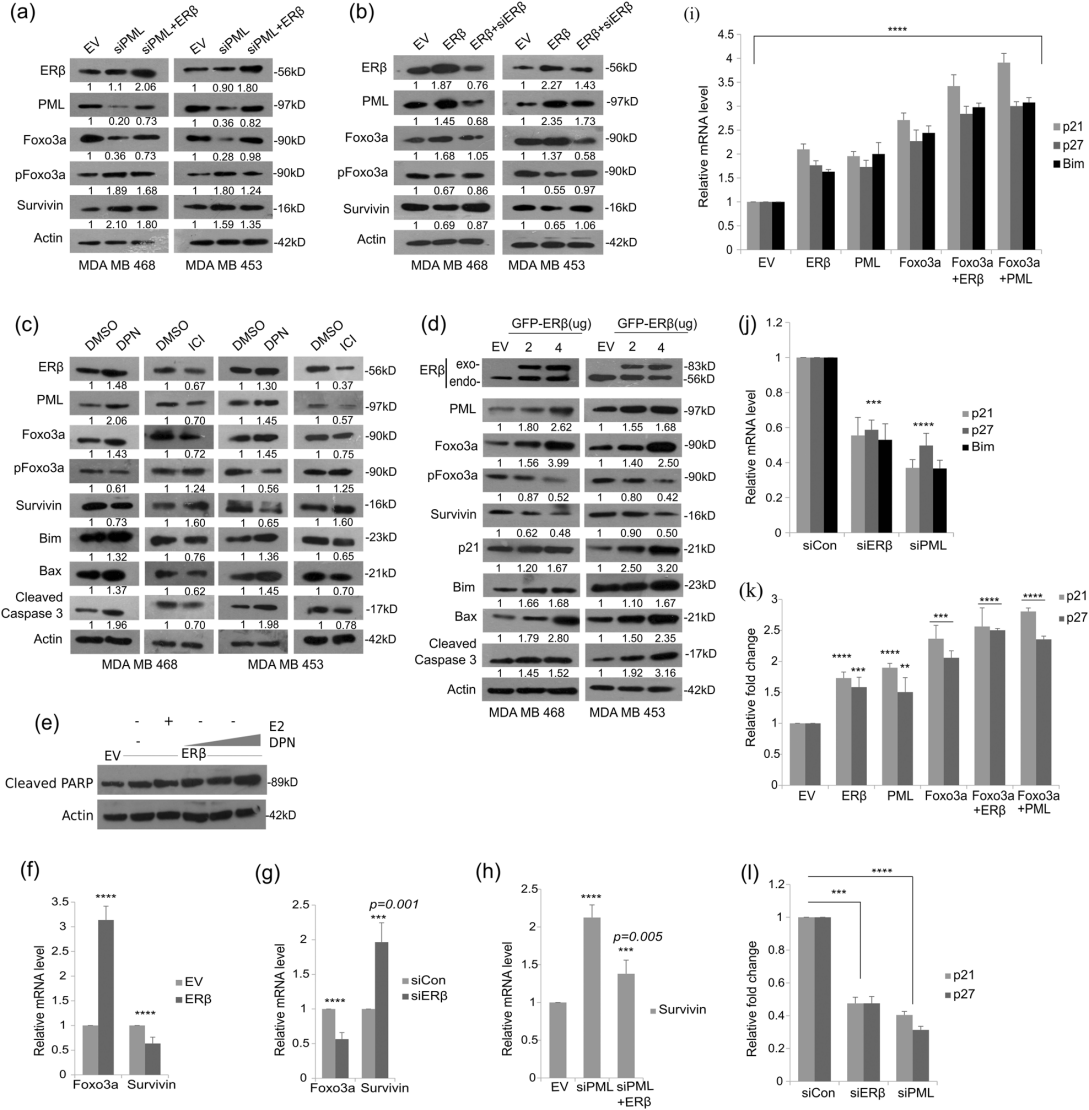
ERβ regulates Foxo3a and Survivin following ‘ERβ-PML’ signaling route in TNBC. **a** Immunoblot analyses of cells transfected with either scrambled siRNA or with siRNA against PML and further transfected with WT ERβ (or its vector) and treated with DPN or DMSO. **b** Immunoblot analyses of cells transfected with WT ERβ, treated with DPN and further transfected with either scrambled siRNA or with siRNA against ERβ. **c** Immunoblot analyses of cells treated with either DPN or ICI. **d** Immunoblot analyses of cells transfected with the empty vector or GFP-ERβ in a dose-dependent manner as indicated, treated with DPN. **e** Immunoblot analyses of MDA MB 468 cells transfected with WT ERβ and treated with either 10 nM E2 (17β estradiol) or increasing doses of DPN (10, 50, and 100 nM). qRT-PCR analysis against Foxo3a and Survivin genes in MDA MB 468 cells transfected with either **f** pcDNA nv5 ERβ or **g** siRNA against ERβ. **h** qRT-PCR analysis against Survivin gene in MDA MB 468 cells transfected with either scrambled siRNA or siRNA against PML and further transfected with WT ERβ or its control vector. **i–j** qRT-PCR analysis of p21^Cip1/Waf1^, p27^Kip1^ and Bim mRNAs performed in MDA MB 468 cells as indicated. **k** Luciferase activity measured in MDA MB 468 cells co-transfected with either pGVB2-p27^Kip1^ promoter or WWP-p21^Cip1/Waf1^-Luc promoter, the indicated DNA constructs and pRL-TK. **l** Luciferase activity measured in MDA MB 468 cells co-transfected with either pGVB2-p27^Kip1^ promoter or WWP-p21^Cip1/Waf1^-Luc promoter, the indicated siRNA constructs and pRL-TK. Data are normalized to Renilla luciferase activity and represented as fold activity with respect to control cells. Error bars represent mean (±) s.d. calculated from three independent experiments. *P* < 0.0001 is represented as **** for highly significant and NS denotes ‘not significant’

We proceeded to assess the role of ERβ-PML signaling on Foxo3a and Survivin. Activated ERβ led to a rise in PML expression from its depleted state under its knocked-down condition. A rescue in PML expression saw a simultaneous rise in Foxo3a with a concomitant decline of pFoxo3a^S253^ and Survivin (Fig. 5a). Furthermore, we depleted already overexpressed and activated ERβ, and observed an initial rise of PML and Foxo3a that reduced under ERβ knockdown conditions. Conversely, pFoxo3a^S253^ and Survivin followed an opposite pattern (Fig. 5b). DPN caused an increase in Foxo3a and other pro-apoptotic factors, Bim, Bax and cleaved Caspase 3 along with a reduction in pFoxo3a^S253^ and Survivin. The converse results were observed under the treatment with ICI (Fig. 5c). These results were further confirmed by conducting a dose dependant overexpression of ERβ aided by ligand binding (Fig. 5d). Additionally, we also see a dose dependent increase in PARP, a notable marker of cellular apoptosis, on ERβ overexpression coupled with increasing doses of DPN (Fig. 5e). The observation at the protein level was faithfully imitated at the transcript level, where ERβ overexpression intensified the expression of Foxo3a and reduced that of Survivin (Fig. 5f). The opposite effect was observed on depletion of endogenous ERβ (Fig. 5g). We were curious to understand whether ERβ mediated down regulation of Survivin was indeed an effect of PML trans-repression. Confirming our hypotheses, inhibition of PML expression was associated with a significant increase in Survivin expression which depleted on the introduction of ERβ (Fig. 5h). If Foxo3a is a bona fide target of ERβ-PML signaling network, the regulator molecules should have a role on the transcriptional activity of Foxo3a. To confirm this, we conducted qRT-PCR studies where overexpression of either ERβ or PML led to an increase in mRNA expression of Foxo3a target genes- p21, p27, and Bim (Fig. 5i). This result is analogous to individual overexpression of Foxo3a itself, where an expected rise in their mRNA expression gets further augmented when the cells were co-transfected with either ERβ or PML. Conversely, knockdown of ERβ and PML leads to a reduction in the gene expression of the Foxo3a target genes (Fig. 5j). Luciferase assays showed that both ERβ and PML upregulated p21 and p27 promoter trans-activation and further augmented the results observed under Foxo3a overexpression (Fig. 5k). Noticeably, upon knockdown of either ERβ or PML, reporter activity of both the promoters declined (Fig. 5l). All these results put together explained that ERβ’s regulation of PML gene is required for the downregulation of anti-apoptotic factor Survivin and increase in the expression and transcriptional activity of tumor suppressor Foxo3a.

### ERβ-PML network causes reduced cell proliferation and migration

**Fig. 6:**
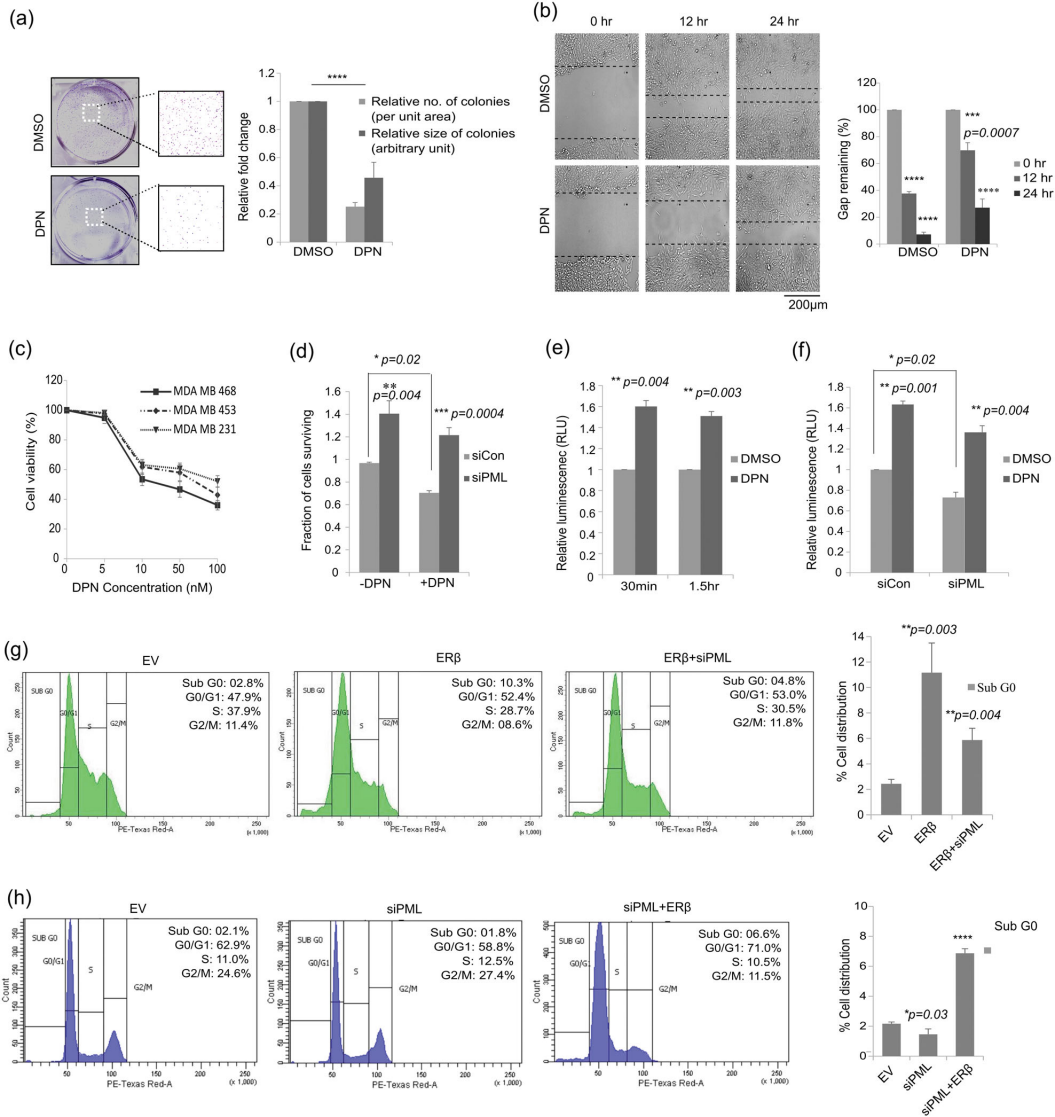
ERβ induction results in decrease in cellular proliferation and migration. **a** Cell proliferation assay measured in MDA MB 468 cells treated with either 10 nM DPN or DMSO kept for 15 days and as observed from colony formation capacity (*n* = 3). The size and number of colonies are represented in the bar diagram. **b** Scratch assay performed on MDA MB 468 cells subjected to DPN or DMSO and at the indicated time slots for a total period of 24 hr. Scale bar of 1000 μm applies to all the images. Percentage of the gap remaining measured is represented as bar diagram. **c** Cell viability as determined by MTT Assay in multiple TNBC cell lines treated with increasing concentration of DPN for 48 hr. **d** MDA MB 468 cells transfected with siCon or siPML and treated with or without 10 nM DPN and cell viability determined by MTT assay as percentage of untransfected cells at the end of 72 hr. **e** Caspase3/7 activity measured in MDA MB 468 cells treated with DPN or DMSO as an increase in luminescence, either 30 min or 1.5 hr post addition of the reagent (mean of three separate experiments and ±s.e.m. calculated using Student’s *t*-test). **f** MDA MB 468 cells transfected with siCon or siPML and treated with or without DPN and Caspase3/7 activity measured 30 min post addition of the reagent (mean of three separate experiments, ±s.e.m. calculated using Student’s *t*-test). **g** Cell cycle distribution measured by flow cytometry in MDA MB 468 cells transfected with exogenous WT-ERβ and further transfected with either scrambled siRNA or with siRNA against PML. **h** Cell cycle distribution measured by flow cytometry in MDA MB 468 cells transfected with either scrambled siRNA or with siRNA against PML and further transfected with WT-ERβ. Error bars represent mean (±) s.d. calculated from three independent experiments. *P* < 0.0001 is represented as **** for highly significant and NS denotes ‘not significant’

ERβ is known to cause a reduced proliferation of TNBC cell lines. Hence, we investigated the effect of ‘ERβ–PML–(Foxo3a/Survivin)’ network on cellular proliferation. A reduction in anchorage dependent growth was observed when cells were treated with DPN as compared to the controls, in colony formation assay (Fig. 6a). The number and the size of colonies formed significantly reduced on DPN treatment. Wound healing assay shows a similar reduction in cellular migration when treated with DPN (Fig. 6b). Cell viability as analyzed from MTT assay is also seen to decrease with an increase in DPN dose where a nominal dose (10 nM) of DPN shows significant reduction in cell viability (Fig. 6c). This effect of ERβ induction was reversed by PML knockdown, as we observed a significant rise in cell survival, as a mark of increased cellular proliferation in siPML transfected cells. DPN stimulation reduced cell survival by 30% in siCon cells but by a lesser proportion (~14%) in siPML cells (Fig. 6d). We further see an increase in Caspase 3/7 activity in the presence of DPN (Fig. 6e), whereas, PML depletion is associated with a reduction in Caspase activity which is rescued upon DPN stimulation (Fig. 6f). Overexpression of ERβ also disturbs the cell-cycle progression as is observed from the increase in the percentage of cells in the Sub-G_0_ and G_0_ stages while a reduction is observed in the G2/M phase. This growth suppressive effect of ERβ is reversed under conditions of PML knockdown, when percentage of cells in the G2/M increases with a drop in the proportion of apoptotic cells (Fig. 6g). The reverse phenomenon is observed when PML depletion causes a rise in the proliferative cells, as also indicated from the viability assay results. Induction of ERβ under these conditions shifts the cell cycle towards apoptosis as observed from a rise in sub-G_0_ population with a concomitant decrease in G2/M phase (Fig. 6h). These results indicate that ERβ promotes growth suppression via apoptosis and these effects are abrogated on PML induction. Thus ERβ induction is important for enhanced expression of PML, leading to activation of tumor suppressors Foxo3a, p21 and caspases, reduction of pro-oncogenic molecule Survivin, and subsequent effect on apoptosis and tumor suppression.

## Discussion

Several lines of research and evidences have elucidated the role of PML in tumor suppression and its concomitant loss in multiple cancers^2,29^. PML expression is reduced or abolished in 21% and 31% of breast carcinomas, respectively, where loss of PML correlates with bad prognosis and increased tumor grade^2^. Putting this literature into our perspective we sought out to understand how PML gene is regulated in breast cancer. A previous work from our group had established the role of ERα in reducing PML protein expression in BCa cells, *via* upregulation of CK2α^31^, of which PML is a notable downstream target^32^. It was our interest to understand how PML expression can be regulated in the absence of ERα and thus be stabilized in BCa cells to bring down oncogenesis. We focussed on ERβ which is gaining prominence for its role in inhibiting the growth and invasiveness of BCa cells.

**Fig. 7:**
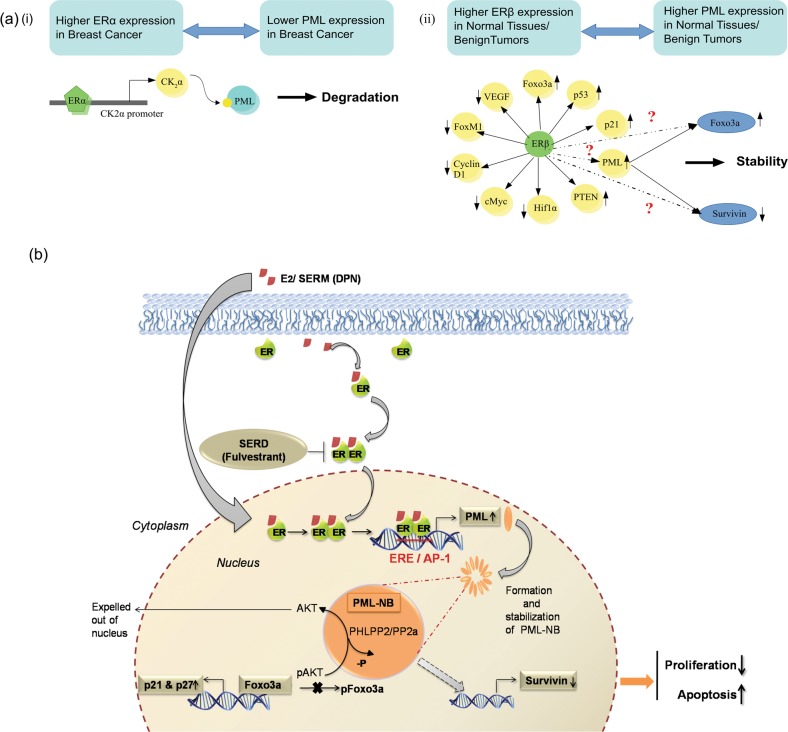
**a** A schematic diagram highlighting PML regulation in breast cancer: (i) Negative correlation between ERα –PML and ERα-CK2α mediated PML degradation in ERα^+^ BCa and (ii) Positive correlation between ERβ-PML and the hypothesis regarding the possibility of ERβ mediated PML gene regulation towards stabilization of PML in ERα^−^ BCa. **b** Model: ERβ follows estrogen signaling, tethering to ERE/AP-1 sites on PML promoter leading to its transcriptional upregulation that allows the formation and stabilization of PML-NBs. PML upregulation leads to subsequent reduction in Survivin and stabilization of Foxo3a and its target genes p21 and p27 and the consequent effect in controlling breast carcinogenesis

ERβ is a transcriptional regulator whereby it up-regulates various tumor suppressors such as p53, p21, and down regulates prominent pro-oncogenic and cell cycle progression factors *viz*., Cyclin D1, cMyc, Hif1α, VEGF, FOXM1 etc^33–37^. We observed that both ERβ and PML maintain a strong positive correlation in human BCa and adjacent normal tissues, with a strong expression in normal tissues that declines in BCa samples. In this context, the possible role of ERβ in regulating PML expression can be studied (Fig. 7a).We observed that ERβ enhances PML mRNA and protein levels in multiple TNBC cell lines. We note that ERβ gets re-localized in discrete nuclear foci once being treated by DPN. Certain previous reports had mentioned the nucleolar localization of ERα and had hypothesized that this might be associated with hormonal induction of pre-ribosomal RNA synthesis^38^ and/or with the promoter of target genes, thus turning them on^39^. This phenomenon of ERβ localization in nucleoli of MDA MB 468 cells is a novel finding and raises pertinent questions regarding its role. Another interesting observation was that unliganded ERβ alone, was able to elicit the regulation of PML, which gets augmented in the presence of DPN. Our luciferase assay results proved our case stronger, where we observed ERβ mediated activation of PML promoter. This is in line with previous reports where unliganded ERβ is capable of interacting with target gene promoters and elicit changes in gene expression^40–44^. It reiterates the thought that transcriptional activity of ERβ is highly cell and promoter dependent and that activity of N-terminal AF-1 might be modulated by several other signaling cascades^45,46^. Here, we are the first to establish and report the presence and the role of ERE and AP-1 sites on PML promoter. ChIP studies indicated a strong binding of ERβ on the PML promoter. Mutation studies proved that ERβ directly associates with PML promoter *via* its DNA binding domain and tethers to both ERE and AP-1 sites. Additionally, ERβ binding on ERE sites provides more impetus for PML promoter activity, as compared to the AP-1 sites. Moreover, ERβ action through AP-1 sites is complicated and is reported to be ligand specific^34,42,47^, further analysis and validation of which would make the study interesting. It was intriguing to decipher what roles a stabilized PML hold in regulating tumor suppression *via* its effector molecules, namely Foxo3a and Survivin. We observed a strong positive correlation of ERβ-PML with Foxo3a and a negative correlation with Survivin, observed both at protein and mRNA levels, in human breast tumors and normal tissues. PML knockdown abolishes Foxo3a expression and causes an increase in expression of Survivin, the results being reversed on introduction of ERβ. A stabilized Foxo3a in lieu of the ERβ-PML network is able to transcriptionally activate its target gene promoters p21 and p27, leading to increase in their expression.

In summary, our work thus establishes a compelling link in controlling oncogenesis where tumor suppressor PML is regulated by a pro-apoptotic molecule ERβ. ERβ binds to estrogen or to several ERβ specific Selective Estrogen Receptor Modulators (SERMs)^47,48^ such as DPN, WAY-202196, WAY-200070, 8b-VE2^49,50^ and activate signaling. On the other hand, Selective Estrogen Receptor Downregulators (SERDs) such as Fulvestrant (ICI 182,780) inhibit receptor dimerization and accelerate ER degradation, thus providing pure anti-estrogenic effect^24,51,52^. Activated ERβ tethers to ERE/AP-1 sites on PML promoter and switches on its transcriptional upregulation. Stabilized PML further controls oncogenesis using bi-fold method: (a) by down-regulating pro-oncogenic Survivin and (b) by acting as a scaffold molecule, causing possible inactivation of active pAKT and thus stabilization of Foxo3a that further activates important cell cycle regulators and markers of tumor suppression: p21 and p27. Thus ERβ-PML network interestingly curbs oncogenesis by inhibition of anti-apoptotic molecule and stabilizing a tumor-suppressor (Fig. 7b). Exploration of this novel ‘ERβ-PML-(Foxo3a/Survivin)’ signaling axis might hopefully provide a new direction in the clinical management of breast cancer. This work can also consider ERβ as a therapeutic target in ERα^−^ tumors thus helping us to develop a new therapeutic network.

## Material and methods

### Human breast tissue samples

Formalin-fixed paraffin-embedded sections, derived from post-surgical human BCa (*n* = 35), TNBC (*n* = 19) and adjacent normal breast (*n* = 24) tissues collected from Indian patients were used in this study. The samples were collected in accordance with all medical and ethical regulations, including patient consent, and with formal approval from the institutional ethical committees of both CSIR-IICB and Park Clinic (source of normal and BCa samples).

### Histological analysis and immunohistochemistry (IHC)

Histological and Immunohistochemical studies were conducted as described before^29,53^. For image scoring purposes, an overall H-score^54^ ranging from 100 to 300 was achieved where the degree of staining (0–100%) was multiplied by intensity pattern of staining (set at 1: negative or weak, 2: moderate and 3: strong). The slides were viewed and images captured at ×200 and ×400 magnifications by EVOS XL Cell Imaging System (Life Technologies-Thermo Fischer Scientific).

### Expression plasmids

ERβ was sub-cloned from pcDNA nv5 vector (purchased from Addgene #22770) to pGZ21dx (referred here as GFP-ERβ) and pcDNA 3.1 (+/+) (referred to as WT-ERβ or ERβ) vectors. Human (−1447 to +250) and mouse (−800 to TSS) PML promoters were PCR amplified using genomic DNA from HEK293 cells and 4T1 cells respectively and cloned into pGL3 basic vector. WWP-p21/Waf1-Luc was purchased from Addgene (#16451) and pGVB2-p27Kip1-Luc promoter was a kind gift to our lab from Dr. Toshiyuki Sakai; Kyoto Prefectural University of Medicine, Japan. pSG5-PML3, encoding PML isoform IV (according to nomenclature established by Jensen, Shiels and Freemont^55^), was a kind gift from Dr. Paul S Freemont; Division of Molecular Biosciences, Imperial College of London, UK. HA-FOXO3a WT was purchased from Addgene (#1787). siRNAs against PML (sc-36284) and ERβ (sc-35325, sc-44297) were purchased from Santa Cruz Biotechnology.

### Cell culture, transfections and treatments

HEK293 and human TNBC cell lines MDA MB 231, MDA MB 468 and MDA MB 453 were maintained in Dulbecco’s Modified Eagle’s Medium (DMEM) and mouse BCa cell line 4T1 in RPMI medium (Invitrogen), supplemented with 10% fetal bovine serum (FBS, Gibco) using standard procedures. Transfections were carried out with either Lipofectamine 2000 (for DNA constructs) or with Lipofectamine RNAimax (for siRNAs) (both of Life Technologies) following manufacturer’s protocol. For transfecting siRNA against ERβ, equal amount of both the siRNAs are used in combination to knock down ERβ. Transfection of plasmids was followed upto 24 hr prior to any addition of drug, while siRNA transfections were performed upto 48 hr.

For estrogen free experiments, the cells were cultured and maintained in phenol red-free DMEM (Invitrogen) supplemented with 5% charcoal stripped FBS (Invitrogen) as described before^31^. The ERβ specific ligand 2,3-Bis(4-hydroxyphenyl) propionitrile (DPN) and ER antagonist ICI 182,780 (Fulvestrant/ICI) were purchased from Sigma and were used at concentration of 10 nM and 1 µM, respectively, for 24 hr, unless otherwise mentioned. Transcriptional inhibitor Actinomycin D (Sigma) was used at a concentration of 10 μg/ml for 4 hr.

### Site directed mutagenesis (SDM) and deletion

Deletions of half ERE sites and AP1 sites in human PML promoter were performed using QuickChange XL Site-Directed Mutagenesis kit (Agilent Technologies)^56^. The mutated promoters were further cloned into pGL3 basic vector. The ERβ DNA binding domain, comprising amino acids 144–225 was deleted and the mutated ERβ DNA was cloned into pcDNA 3.1 vector. All the constructs were verified by sequencing. Sequences of the primers are given in Additional file 1.

### Immunblotting (IB)

Preparation of whole cell lysates and IB analyses were performed as described before^57^. The following primary antibodies were used: ERβ (sc-8974), PML (sc-5621), p21 (sc-53870), Actin (sc-1616), and GFP (sc-9996) (SantaCruz Biotechnology); Foxo3a (ab53287), p-Foxo3a (S253)(ab31109) (Abcam); Survivin (#2808), Bim (#2819), Bax (#2772), cleaved PARP (#9625) and cleaved Caspase 3 (#9664) (Cell Signalling Technology); HRP-tagged anti-rabbit and anti-mouse secondary antibodies (Cell Signalling Technology); HRP-tagged anti-goat secondary antibody (Sigma-Aldrich). Densitometry values of the immunoblots were computed using GelQuant.Net software.

### RNA preparation and quantitative real time PCR

Total RNA was extracted by using Trizol reagent (Invitrogen) as per the manufacturer’s protocol. cDNA was prepared and Real-time PCR performed by using FastSYBR Green Master Mix (Applied Biosystems) in Via7 Real-Time PCR Instrument (Applied Biosystems) as described before^56^. Sequences of the primers are given in Additional file 1.

### Luciferase assay

Luciferase assays were performed as described before^58^. All experiments for luciferase assays were followed upto 48 h post transfection and 24 hr for any additional drug treatment. A minimum of three biological repeat experiments along with three technical repeats each were conducted to empirically determine the quantifications.

### Chromatin Immunoprecipitation (ChIP) Assay

ChIP assay was conducted as described previously^59^. In brief, the cells were subjected to crosslinking with 0.8% formaldehyde, reaction quenched with 125 mM glycine and further sonicated to an average size of 500–800 bp. In total 10% of pre-cleared sonicated chromatin (unless otherwise mentioned) was kept aside as Input and the rest was incubated overnight with 2 μg each of primary antibodies against either ERβ, Polymerase II or normal rabbit IgG (purchased from Cell Signaling Technologies) and further pulled-down with pre-blocked protein A Sepharose beads. The precipitated chromatin was eluted from the beads and de-crosslinked. DNA was purified from immunoprecipitated chromatin fragments and PCR amplified using Qiagen’s Top Taq master mix. The PCR products were either run on a 1% agarose gel (for endogenous ERβ binding), or subjected to a quantitative RT-PCR using SYBR Green master mix. PCR cycling conditions are as follows: one cycle at 94 °C for 5 min, 40 cycles at 95 °C for 30 s, 54.4 °C for 30 s, and at 72 °C for 30 s, followed by one final cycle at 72 °C for 10 min.

### Cell viability and wound healing assays

Cell viability assay using MTT and wound healing (scratch) assay on 70–80% confluent monolayer cells were conducted as described before^57^.

### Survival assay

The cells (2.5 × 10^3^) in triplicate were treated with DMSO or DPN for 24 hr in 5% charcoal stripped serum containing media. After treatment, the cells were grown for another 15 days in complete medium. Assay was performed as described before^60^.

### Caspase3/7 assay

MDA MB 468 cells were plated in 96-well plates (in triplicate), and treated with DMSO or DPN for 24 hr. Caspase-Glo 3/7 Reagent(Caspase-Glo® 3/7 Assay, Promega) was added to the wells and luminescence recorded after 30 min and also after 1.5 hr, following manufacturer’s instructions.

### Immuno-fluorescence microscopy

MDA MB 468 cells were seeded over sterile cover slips placed inside 35 mm tissue culture dishes and cultured as per prescribed condition, or as mentioned above, to a confluency of 60%. The cells were harvested as follows: 4% paraformaldehyde fixation, 0.5% Triton-X-100 permeabilization and blocking with 2.5% BSA in PBS. Standard protocol of immuno-staining was followed and the cells were stained with primary antibodies, ERβ (Abcam ab288) and PML (Abcam ab53773) and fluorochrome-conjugated secondary antibodies (Alexa-Fluor 488 or 594). 4, 6-diamino-2-phenylindole (DAPI) was used as nuclear counter stain. All slides were viewed and images captured at ×120 using FluoView FV10i confocal laser scanning microscope (Olympus Life Science).

### Cell cycle analysis

MDA MB 468 cells were transfected as mentioned in the figures for the requisite durations as mentioned above, and harvested using Trypsin. The cells were ethanol (70%) fixed and further processed for cell-cycle analysis as described before^57^and analyzed in BD LSR-Fortessa using FACS-Diva software (BD Biosciences).

### Statistical analyses

Paired Student’s *t*-test was employed to determine the significance value in all experiments. The significance is presented as *P 0.05, **P 0.005 and ***P 0.001, and non-significant differences are presented as *NS*. The differences in H-score values of all the concerned proteins between normal breast and BCa tissues were analyzed by Mann–Whitney U-test. All statistical analyses were performed using either SPSS (IBM) or GraphPad QuickCals software packages.

## Supplementary information

Additional File-1

Additional File-2

## Data Availability

The data supporting the conclusion of this study are included within this manuscript and the Supplementary files.
