# Supplementary material for: Promyelocytic Leukemia (PML) gene regulation: implication towards curbing oncogenesis
Source: Cell Death Dis. 2019 Sep 10;10(9):656. doi: 10.1038/s41419-019-1889-2 (PMC6736969; doi:10.1038/s41419-019-1889-2)
Supplement: Supplementary file 1 — Additional File-1 [file 41419_2019_1889_MOESM1_ESM.pdf]

Additional File-1: Primers and oligonucleotide sequences used in this study

Name	Sequence (5'-3')
Cloning and Sub-cloning	
PML promoter (human):	F- AATCTCGAGACCTGAATGCAACTCGTGAA<br>R- AATAAGCTTCCATCATCCCCTAACCCAATAG
PML promoter (mouse):	F- AATCTCGAGGTTCTGTCACCACGGTCCTT<br>R- AATAAGCTTGCCAGAAAACTGAGCGAGAG
ERβ(Δ 144-225):	F- ATGAATATCCAGCCATGACATTCCAAATGTGTTGTGGCC<br>R- GGCCACAACACATTTGGAATGTCATGGCTGGATATTCAT
qRT-PCR	
18s rRNA	F- GCTTAATTTGACTCAACACGGGC<br>R- AGCTATCAATCTGTCAATCCTGTC
ERβ (human)	F- AAGAATATCTCTGTGTCAAGGCCATG<br>R- GGCAATCACCCAAACCAAAG
ERβ (mouse)	F- TACACTGATTCGTGGCTGGA<br>R- TTACGGTGTCTGGTCCTGTG
PML 1 (human)	F-GATGGCTTCGACGAGTTCAA<br>R-GGGCAGGTCAACGTCAATAG
PML 2 (human)	F-GATCTCTGCGCTGATGTCG<br>R-AACATCTTCTGCTCCAACCC
PML (mouse)	F- AGCAGGAGGCTTCTCAGACAGT<br>R- CTTGATGATCTTCCTGGAGCAA
P21 (human)	F-GCAGACCAGCATGACAGATTT<br>R-GGATTAGGGCTTCCTCTTGGA
P21 (mouse)	F- ATCACCAGGATTGGACATGG<br>R- CGGTGTCAGAGTCTAGGGGA
Foxo3a (human)	F-TGCTAAGCAGGCCTCATCTC<br>R-CTTGTGTCAGTTTGAGGGTCT
Foxo3a (mouse)	F- CTTCATTCTGAACGCGCA<br>R- CTTCAAGGATAAGGGCGACA
p27	F-TAATTGGGGCTCCGGCTAACT<br>R-TTGCAGGTCGCTTCCTTATTC
Bim	F- CTGCTGTCTCGATCCTCCAGT<br>R- GTCGTAAGATAACCATTCGTG
Bax	F- CTTTTGCTTCAGGGTTTCATC<br>R- TTGAGACACTCGCTCAGCTTC
Survivin	F- ACCACCGCATCTCTACATTCA<br>R- CTCGTTCTCAGTGGGGCAGT

qRT-PCR/PCR (ChIP)	
hPML1	F- CTCTGCCTCCTCAGCCAATC<br>R- GCCTGGCTCTTGTAAACCTT
hPML2	F- GGAACAGAGTACGGAATGGGT<br>R- TGGAAAGGCTGGTGGATCT
hPML3	F- TCAGATCCACCAGCCTTTCC<br>R- TTGGAACCAGGGAACAACAC
hPML4	F- AGTGCCAGTGTGAACGGATG<br>R- CAGTTGGCTGAGTCCCTTGA
mPML1	F- TTACTTCTCATAGGCCACCTGC<br>R- GAAGGGTTTCTTACAGCGTGTC
mPML2	F- CAGACCTCCACTCCTTTCCA<br>R- TACTGGGATGGGTTTGCCTG
Actin	F-TGCACTGTGCGGCGAAGC<br>R- TCGAGCCATAAAAGGCAA
GAPDH	F- CGGCTACTAGCGGTTTTACG<br>R- GGCTGCGGGCTCAATTTAT
Neg Control	F-GGGGGATCAGATGACAGTAAA<br>R-AATGCCAGCATGGGAAATA
Site Directed Mutagenesis	
PML-prom-ΔERE1:	F- TTCCCTCCAGCCTGTACTCCCAGGCC<br>R- GGCCTGGGAGTACAGGCTGGAGGGAA
PML-prom-ΔERE2:	F- AGCCGGGGCGCCAGTGAGGTCAC<br>R- GTGACCTCACTGGCGCCCCGGCT
PML-prom-ΔERE3:	F- AGTTCCAGGTCACTGGGTCAGCGCCC<br>R- GGGCGCTGACCCAGTGACCTGGAACT
PML-prom-ΔAP1:	F- CCTGCTCCCTCACCTGGCTTCCCAGG<br>R- CCTGGGAAGCCAGGTGAGGGAGCAGG
PML-prom-ΔAP2:	F- ATCAGCTGTGCATGATGCTCTGACAACCTAGGAG<br>R- CTCCTAGGTTGTCAGAGCATCATGCACAGCTGAT
PML-prom-ΔAP3:	F- CTGCAGACCTACCGCGTAAAGCGGGAGAGG<br>R- CCTCTCCCGCTTTACGCGGTAGGTCTGCAG
