# Supplementary material for: Promyelocytic Leukemia (PML) gene regulation: implication towards curbing oncogenesis
Source: Cell Death Dis. 2019 Sep 10;10(9):656. doi: 10.1038/s41419-019-1889-2 (PMC6736969; doi:10.1038/s41419-019-1889-2)
Supplement: Supplementary file 2 — Additional File-2 [file 41419_2019_1889_MOESM2_ESM.pdf]

Supplementary Figures

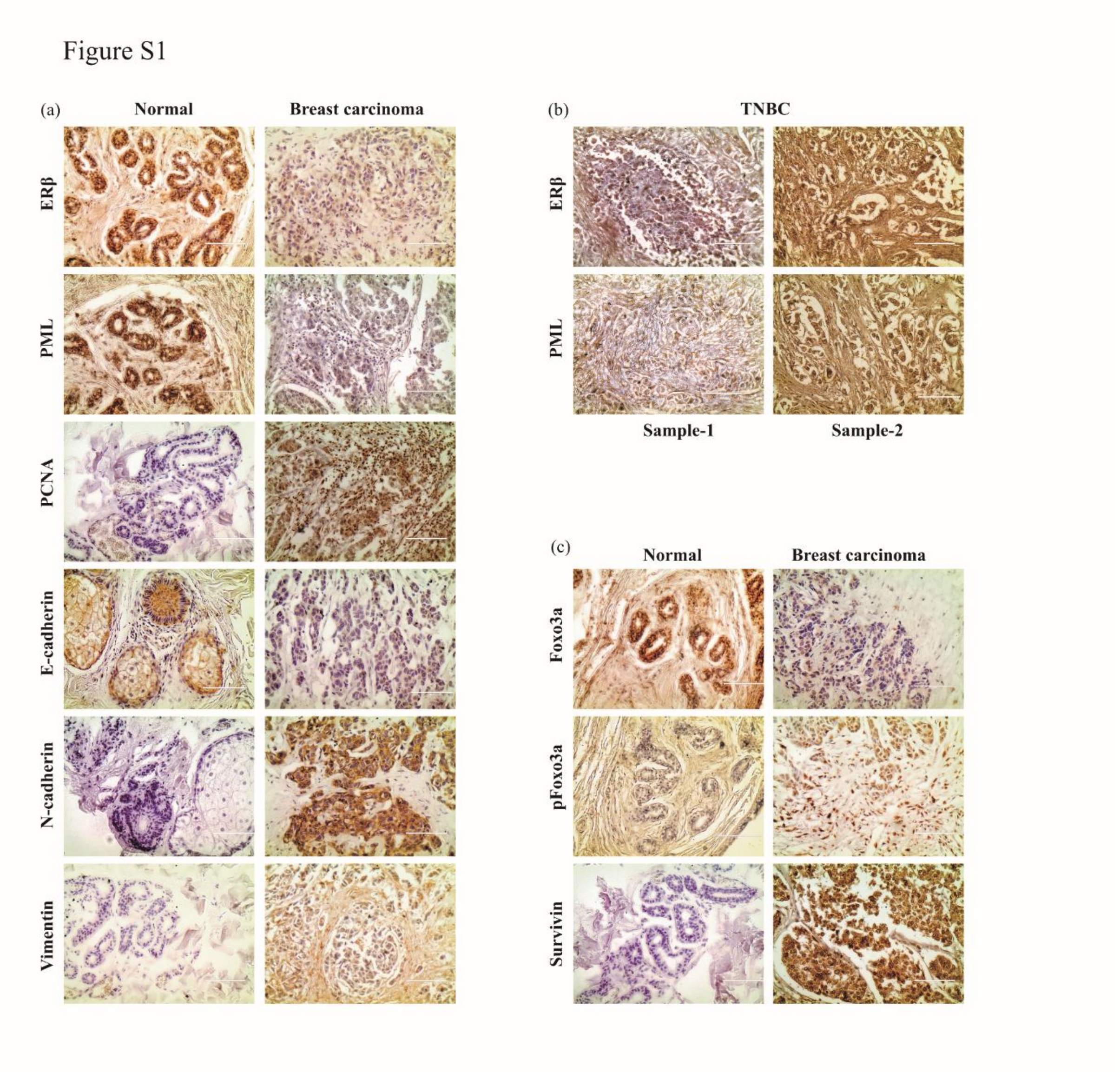

Figure S2

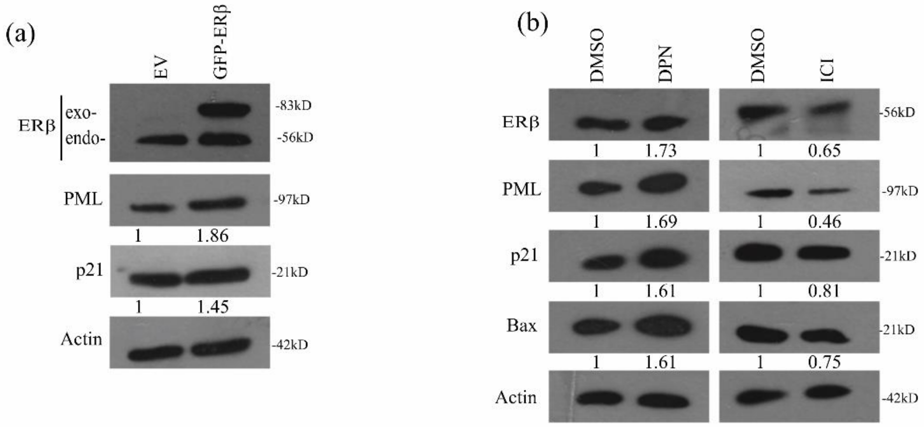

(a)
EV
GFP-ERβ
ERβ
exo-
endo-
-83kD
-56kD
PML
-97kD
1
1.86
p21
-21kD
1
1.45
Actin
-42kD
(b)
DMSO
DPN
DMSO
ICI
ERβ
-56kD
1
1.73
1
0.65
PML
-97kD
1
1.69
1
0.46
p21
-21kD
1
1.61
1
0.81
Bax
-21kD
1
1.61
1
0.75
Actin
-42kD

Figure S3

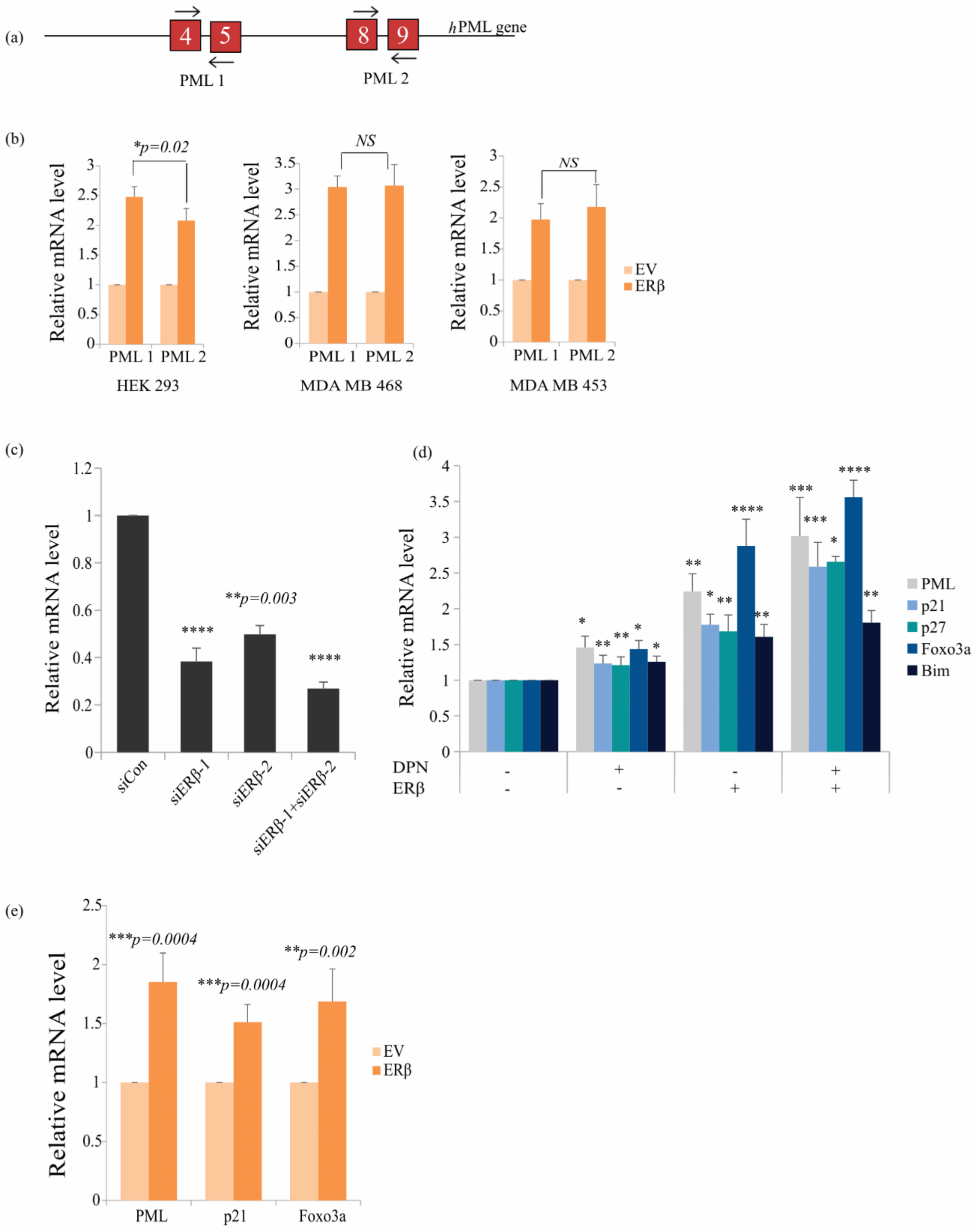

(a)
4
5
8
9
*h*PML gene
PML 1
PML 2
(b)
*p=0.02
NS
NS
Relative mRNA level
PML 1 PML 2
HEK 293
MDA MB 468
MDA MB 453
EV
ERβ
(c)
Relative mRNA level
**p=0.003
siCon
siERβ-1
siERβ-2
siERβ-1+siERβ-2
(d)
Relative mRNA level
PML
p21
p27
Foxo3a
Bim
DPN
ERβ
(e)
Relative mRNA level
***p=0.0004
***p=0.0004
**p=0.002
EV
ERβ
PML
p21
Foxo3a

Figure S4

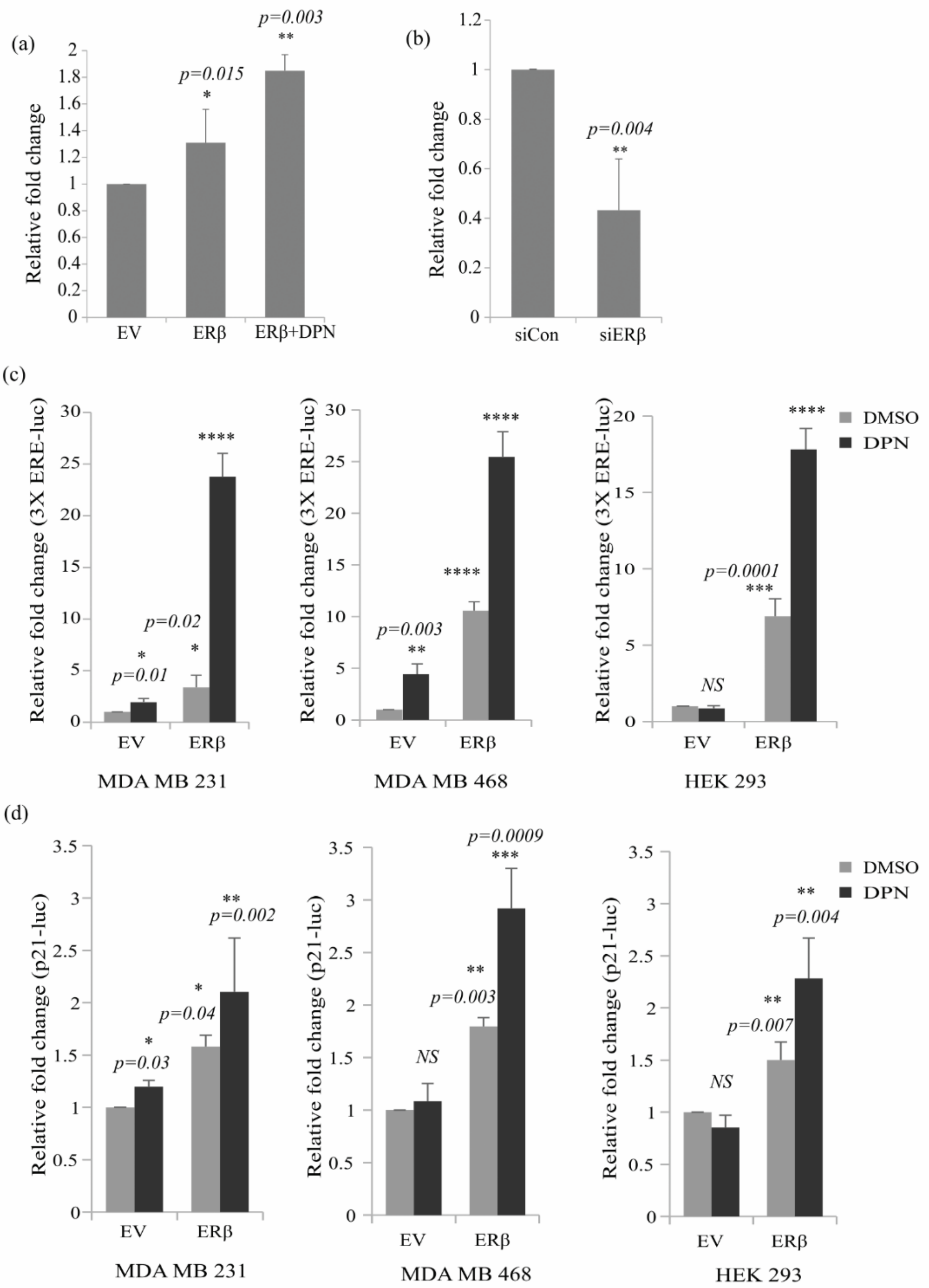
(a)
Relative fold change
EV
ERβ
ERβ+DPN
p=0.015
*
p=0.003
**
(b)
Relative fold change
siCon
siERβ
p=0.004
**
(c)
Relative fold change (3X ERE-luc)
DMSO
DPN
p=0.01
*
p=0.02
*
****
p=0.003
**
****
****
NS
p=0.0001
***
****
EV
ERβ
MDA MB 231
MDA MB 468
HEK 293
(d)
Relative fold change (p21-luc)
p=0.03
*
p=0.04
*
**
p=0.002
NS
p=0.003
**
p=0.0009
***
NS
p=0.007
**
p=0.004
**
EV
ERβ
MDA MB 231
MDA MB 468
HEK 293

Figure S5

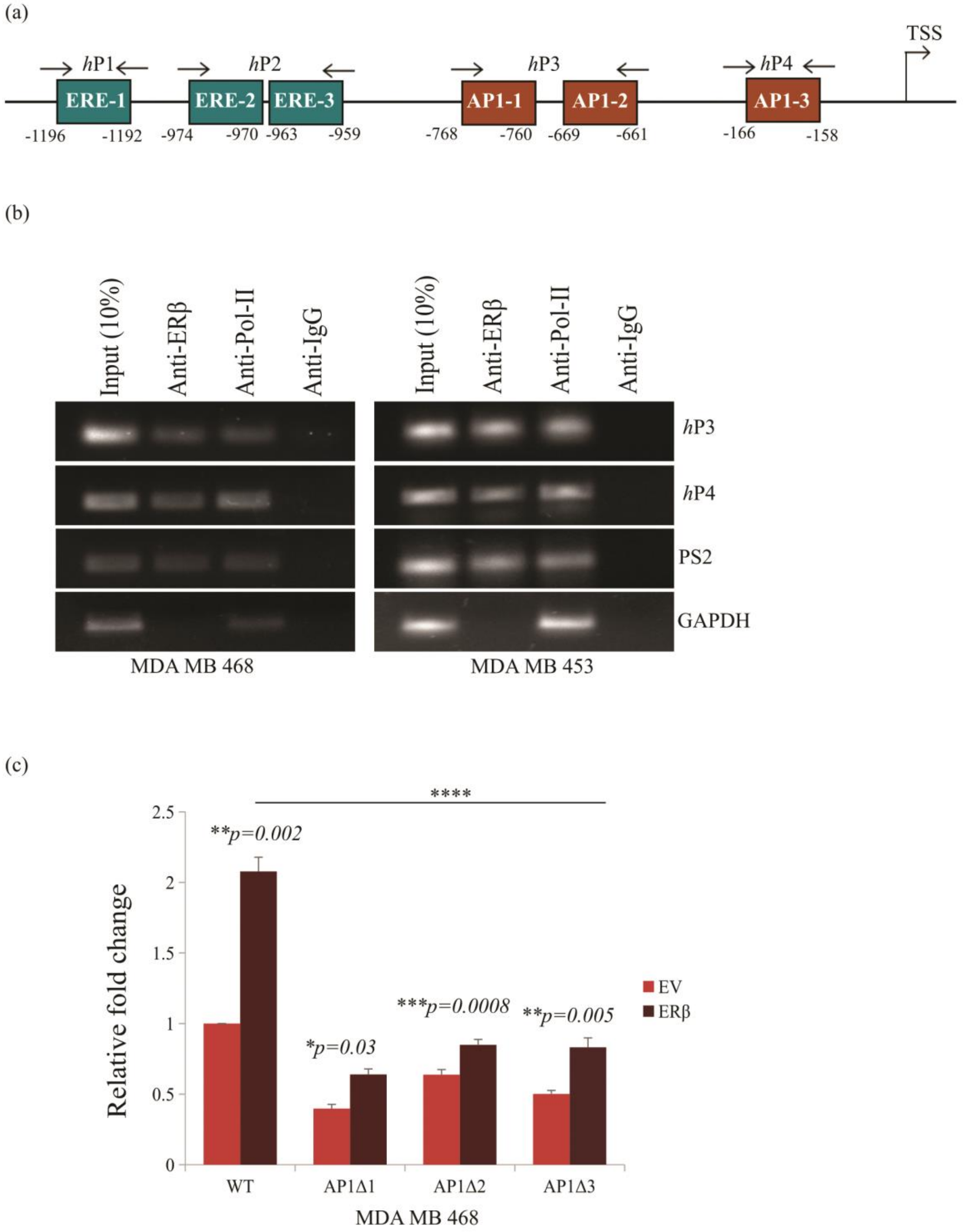

(a)
TSS
hP1
hP2
hP3
hP4
ERE-1
ERE-2
ERE-3
AP1-1
AP1-2
AP1-3
-1196
-1192
-974
-970
-963
-959
-768
-760
-669
-661
-166
-158
(b)
Input (10%)
Anti-ERβ
Anti-Pol-II
Anti-IgG
hP3
hP4
PS2
GAPDH
MDA MB 468
MDA MB 453
(c)
****
**p=0.002
*p=0.03
***p=0.0008
**p=0.005
Relative fold change
2.5
2
1.5
1
0.5
0
WT
AP1Δ1
AP1Δ2
AP1Δ3
MDA MB 468
EV
ERβ

Supplementary Figure Legends

Figure S1 | Concomitant loss of ERβ and PML expression in breast cancer. a-c Representative IHC images of the candidate proteins depicting the staining pattern/localization and intensities, in human BCa and adjacent normal breast tissue. All images are taken at 400X magnification.

Figure S2 | ERβ positively regulates PML protein expression in HEK293 cells. a Immunoblot analyses of cells transfected with GFP-ERβ or the empty vector and further treated with DPN. **b** Immunoblot analyses of cells treated with either DPN (10nM) or ICI (1uM) or DMSO control.

Figure S3 | ERβ regulates PML gene expression. **a** Schematic representation of two human PML RT-PCR primer sets designed that spans over two different exon-exon junctions. **b** qRT-PCR analysis of PML mRNA performed in indicated cells transfected with GFP-ERβ and analysed with two different PML RT-PCR primer sets. Figures represent normalized relative mRNA fold change with respect to 18s rRNA. **C** qRT-PCR analysis of ERβ mRNA in MDA MB 468 cells transfected with 2μg of siERβ1 or siERβ2 either individually or in combination. Equal amount of siCon was used to balance the plasmid gain. **d** qRT-PCR analysis performed on HEK293 cells transfected with GFP-ERβ and/or treated with DPN. **e** qRT-PCR analysis performed on 4T1 cells transfected with GFP-ERβ. Error bars represent mean (±) s.d. calculated from three independent experiments. *=p<0.01, **=p<0.001, ***=p<0.0001, ****=p<0.0001. *NS*, denotes 'Not Significant'

Figure S4 | ERβ regulates PML promoter activity. **a** Luciferase activity measured in HEK293 cells co-transfected with WT-ERβ, pGL3-PML-prom and Renilla luciferase construct

pRL-TK and further treated with either DMSO or DPN. The figure represents relative fold change in luciferase readings, normalized against Renilla reporter activity. **b** Luciferase activity measured in HEK293 cells co-transfected with either scrambled siRNA or siRNA against ERβ, pGL3-PML-prom and pRL-TK. Luciferase activity measured in the mentioned cells, co-transfected with WT-ERβ/empty vector and Renilla luciferase construct pRL-TK along with **c** 3X-ERE-TATA-luc or **d** WWP-Luc (p21/WAF1 promoter) and further treated with either DMSO or DPN. Error bars represent mean (±) s.d. calculated from three independent experiments. P<0.0001is represented as ****for highly significant. *NS*, denotes 'Not Significant'

Figure S5 | ERβ feebly interacts with chromatin at the AP1 sites to regulate PML promoter activity. **a** Schematic representation of human PML promoter designed -1447 to +250 bp relative to TSS that harbours 3 ERE and 3 AP1 sites and can be potentially responsible for ERβ mediated PML gene expression. **b** Chromatin immunoprecipitation (ChIP) assay was performed using the indicated antibodies in human MDA MB 468 and MDA MB 453 cell lines. RNA Polymerase II (Pol II) and IgG served as positive and negative controls respectively and PS2 promoter served as the control for ERβ binding. The immunoprecipitated DNAs were PCR amplified using primers (*h*P3, *h*P4 for human PML to amplify the AP-1 sites) designed as depicted in the figure. **c** Three mutant human PML promoters were constructed by deleting one of the three AP-1 sites, one at a time. Luciferase activity measured on transfected MDA MB 468 cells with either WT-ERβ along with pGL3-WT-PML-prom or its deletion constructs with pRL-TK. Data are normalized to Renilla luciferase activity and represented as fold activity with respect to control cells. Error bars represent mean (±) s.d. calculated from three independent experiments. P<0.0001is represented as ****for highly significant.
